# Association between Breastfeeding and Endometrial Cancer Risk: Evidence from a Systematic Review and Meta-Analysis

**DOI:** 10.3390/nu7075248

**Published:** 2015-07-14

**Authors:** Lianlian Wang, Jingxi Li, Zhan Shi

**Affiliations:** Department of Obstetrics, The Fourth Affiliated Hospital of China Medical University, Shenyang 110032, China; E-Mails: jingxili888@yeah.net (J.L.); zhanshi888@yeah.net (Z.S.)

**Keywords:** breastfeeding, endometrial cancer, dose-response, meta-analysis

## Abstract

Quantification of the association between breastfeeding and risk of endometrial cancer is still conflicting. We therefore conducted a meta-analysis to assess the association between breastfeeding and endometrial cancer risk. Pertinent studies were identified by a search of PubMed and Web of Knowledge through April 2015. A random effect model was used to combine the data for analysis. Sensitivity analysis and publication bias were conducted. Dose-response relationships were assessed by restricted cubic spline and variance-weighted least squares regression analysis. Fourteen articles involving 5158 endometrial cancer cases and 706,946 participants were included in this meta-analysis. Pooled results suggested that breastfeeding significantly reduced the risk of endometrial cancer (summary relative risk (RR): 0.77, 95% CI: 0.62–0.96, I^2^: 63.0%), especially in North America (summary RR: 0.87, 95% CI: 0.79–0.95). A linear dose-response relationship was found, with the risk of endometrial cancer decreased by 2% for every one-month increase in the duration of breastfeeding (summary RR: 0.98, 95% CI: 0.97–0.99). Our analysis suggested that breastfeeding, particularly a longer duration of breastfeeding, was inversely associated with the risk of endometrial cancer, especially in North America, but not in Europe and Asia, probably due to the small number of cases included. Due to this limitation, further studies originating in other countries are required to assess the association between breastfeeding and endometrial cancer risk.

## 1. Introduction

Incidence of endometrial cancer has been increasing worldwide [1]. Endometrial cancer is the most common malignancy of the female genital tract and the fourth most common malignancy in women, after breast, lung and colorectal cancers [2]. The risk for endometrial cancer is related to stimulation of the endometrium by estrogen, as explained by the “unopposed estrogen hypothesis” [3]. Thus, endometrial cancer risk is increased in women who have a high level of plasma estrogen that is unopposed by progesterone [4,5]. Therefore, breastfeeding might also contribute to the decreased risk for endometrial cancer, because estrogen is opposed by progesterone during breastfeeding. A number of epidemiologic studies have been published to explore the relationship between breastfeeding and endometrial cancer risk. However, the results are inconsistent. In this study, we conducted a meta-analysis to (1) first assess the association between breastfeeding and endometrial cancer risk; (2) assess the dose-response association between breastfeeding and endometrial cancer risk for every one-month increment of breastfeeding; and (3) assess the heterogeneity and publication bias among studies.

## 2. Methods

### 2.1. Search Strategy

We performed a literature search up to April 2015 using the databases of PubMed [6] and Web of Knowledge [7], using the following search terms: (breastfeeding OR breast feed OR reproductive factors OR lactation OR infant nutrition OR breast milk OR milk human) AND (endometrial) AND (cancer OR neoplasm OR carcinoma OR tumor). Two investigators searched articles and reviewed all retrieved studies independently.

### 2.2. Study Selection

For inclusion, studies should fulfill the following criteria: (1) have a prospective or retrospective design; (2) the exposure of interest were the association between ever breastfeeding (the women with a history of breastfeeding) *vs.* never breastfeeding or the total duration of breastfeeding; (3) the outcome of interest was endometrial cancer; (4) relative risk (RR) or odds ratio (OR) with a 95% confidence interval (CI) was provided (we presented all results with RR for simplicity); and (5) for dose-response analysis, the duration of breastfeeding for each category must also be provided.

### 2.3. Data Extraction

Data extraction was carried out by two reviewers. Disagreements were discussed and resolved by a third reviewer. Data abstracted from each study were as follows: the first author’s last name, year of publication, study region and design, study sample size (number of cases and controls or cohort size), range of follow-up for prospective studies, exposure and outcome assessment including ever breastfeeding and the total or average breastfeeding duration categories, the RR and 95% CI for ever breastfeeding compared with never breastfeeding and longest category compared with shortest category of breastfeeding, and factors adjusted for in the individual study. If multiple estimates of the association were available, we abstracted the estimate that adjusted for the most covariates.

#### Quality Assessment

The quality of studies was examined and controlled in accordance with checklists of Preferred Reporting Items for Systematic reviews and Meta-Analyses for randomized trials (PRISMA) [8]. To determine the quality score of included studies, two reviewers independently performed the quality assessment by using the Newcastle–Ottawa Scale [9], which is a validated scale for non-randomized studies in meta-analyses [10]. The Newcastle–Ottawa Scale is a nine-point scale that allocates points based on the selection process of cohorts (0–4 points), the comparability of cohorts (0–2 points), and the identification of the exposure and the outcomes of study participants (0–3 points). We assigned scores of 0–3, 4–6, and 7–9 for low, moderate, and high quality studies, respectively.

### 2.4. Statistical Analysis

This dose-response meta-analysis was performed using the method proposed by Greenland and Longnecker [11] and Orsini *et al.* [12], which takes into account the correlation between the log RR estimates across breastfeeding. A random-effects model was used to combine study-specific RR (95% CI). We also explored the possibility of nonlinear relationships by modeling duration of breastfeeding using restricted cubic splines with three knots at the 25th, 50th and 75th percentiles of breastfeeding duration. A *p*-value for nonlinearity was calculated by testing against the null hypothesis that the coefficient of the second spline is equal to 0 [13]. The method requires that the distribution of cases and person-years or participants and the RR with the variance estimates for at least three quantitative exposure categories are known. When the cases and person-years were not available, we estimated the slopes (linear trends) by using variance-weighted least squares regression analysis [14,15]. The median duration of breastfeeding for each specific category was assigned to each corresponding log RR estimate. If the median duration was not reported in the article, we used the midpoint between the upper and lower boundary. If the lowest category was open-ended, its lower boundary was set to zero. If the upper boundary of the highest category was left unspecified, we assumed the category to be of the same amplitude as the preceding one. Statistical heterogeneity across studies was assessed using the Q and I^2^ statistics [16]. An I^2^ values of <30%, 30%–75% and >75% represent low, moderate and high heterogeneity, respectively. Meta-regression was performed to assess the potentially important covariates that might exert substantial impact on between-study heterogeneity [17]. A sensitivity analysis was performed with one study removed at a time to assess whether the results could have been affected markedly by a single study [18]. Publication bias was evaluated using Egger regression asymmetry test [19].

All statistical analyses were conducted with STATA version 10.0 (StataCorp LP, College Station, TX, USA). Two-tailed *p* ≤ 0.05 was accepted as statistically significant.

## 3. Results

### 3.1. Study Characteristics

**Figure 1:**
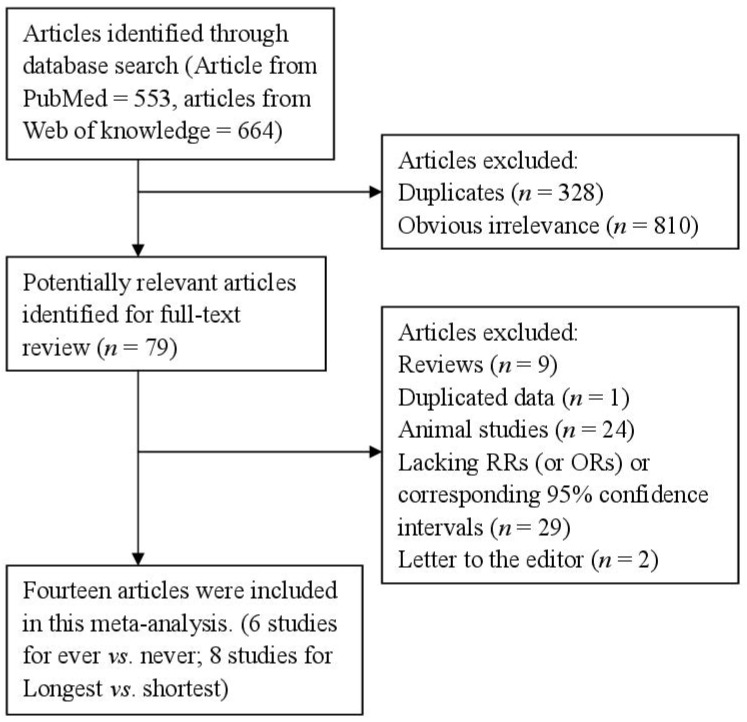
The flow diagram of screened, excluded, and analyzed publications.

After the search strategy, 553 articles from PubMed and 664 from the Web of Knowledge were searched, and 79 articles were reviewed in full after reviewing the title/abstract. Sixty-five of these 79 articles were subsequently excluded from the meta-analysis for various reasons. In total, 14 articles [20,21,22,23,24,25,26,27,28,29,30,31,32,33] (four prospective studies and 10 retrospective studies) involving 5158 endometrial cancer cases and 706,946 participants were used in this meta-analysis. The detailed steps of our literature search are shown in Figure 1. The characteristics of these studies are presented in Table 1. Six studies were conducted in North America, four in Asia, three in Europe and one was a mix-population study. The quality of studies was generally good; with results of study quality assessment yielded a score of 6 or above for all included studies, with an average score of 7.2.

**Table 1:** Characteristics of studies on breastfeeding and endometrial cancer risk.

First Author, Year	Country	Study Design	Cases, Age	Category	RR (95% CI)	Adjustment or Matched for
Brinton *et al.*, 1992, [20]	United States	Retrospective	405, 20–74	Ever *vs.* never	1.01 (0.60–1.60)	Adjusted for age at interview, years of education, recent weight, oral contraceptive use, and menopausal estrogen use.
Brinton *et al.*, 2007, [21]	Polish	Retrospective	551, 20–74	≥24 months *vs.* never	0.72 (0.40–1.20)	Adjusted for age, study, site, years of education, age at menarche, number of full-term births, ever use of oral contraceptives, ever use of oral hormones, ever smoking, recent body mass index.
Dossus *et al.*, 2009, [22]	Denmark, France, Germany, Greece, Italy, The Netherlands, Norway, Spain, Sweden and United Kingdom	Prospective	1017, 30–80	>18 months *vs.* ≤1 months	0.77 (0.54–1.11)	Adjusted for age and center stratified and adjusted for BMI, physical activity, alcohol, diabetes, smoking status and education.
Elwood *et al.*, 1977, [23]	United States	Retrospective	410, 55–59	Ever *vs.* never	1.0 (0.7–1.5)	Na.
Herrinton *et al.*, 2001, [24]	United States	Retrospective	280, 20–54	Ever *vs.* never	0.95 (0.65–1.40)	Adjusted for history of oral contraceptive use and educational attainment.
Hirose *et al.*, 1999, [25]	Japan	Retrospective	133, 30–80	>12 months *vs.* 1–5 months	1.48 (0.63–3.49)	Adjusted for age and body mass index.
Newcomb *et al.*, 2000, [26]	United States	Retrospective	586, 40–79	>24 months *vs.* never	0.84 (0.52–1.40)	Adjusted for age, smoking status, education, body mass, postmenopausal hormone therapy, and parity.
Okamura *et al.*, 2006, [27]	Japan	Retrospective	155, 20–80	Ever *vs.* never	0.37 (0.17–0.82)	Adjusted for age, BMI, oral contraceptive use.
Rosenblatt *et al.*, 1995, [28]	Australia, Israel, Chile, China, Philippines, and Thailand	Retrospective	136, 20–75	>72 months *vs.* never	0.23 (0.08–0.68)	Adjusted for number of pregnancies and age at menarche.
Salazar-Martinez *et al.*, 1999, [29]	Mexico	Retrospective	85, 20–75	>25 months *vs.* never	0.33 (0.17–0.65)	Adjusted by age, hormonal use, number of pregnancies, smoking, diabetes mellitus, hypertension, physical activity, menopausal status, and body build index.
Sugawara *et al.*, 2013, [30]	Japan	Prospective	32, 40–79	Ever *vs.* never	0.31 (0.12–0.81)	Adjusted for age, BMI, family history of cancer, education, job status, smoking status, alcohol consumption, time spent walking, total calorie intake, menopausal status, age at menarche, age at first delivery, number of deliveries, history of oral contraceptive drug use, and history of hormone replacement therapy.
Wernli *et al.*, 2006, [31]	China	Prospective	206, 30–80	>36 months *vs.* ≤1 months	0.62 (0.35–1.09)	Adjusted for age at baseline and number of live births.
Xue *et al.*, 2008, [32]	United States	Prospective	708, 30–55	>9 months *vs.* never	0.99 (0.77–1.29)	Adjusted for age, premature birth, birth order, birth weight, family history of endometrial cancer, age at menarche, oral contraceptive use, parity, age at first birth, age at last birth, physical activity, cigarette smoking, tamoxifen use, menopausal status, age at menopause, postmenopausal hormone use, BMI, BMI at age 18 years, and somatotype at ages 5 and 10 years.
Zucchetto *et al.*, 2009, [33]	Italy	Retrospective	454, 18–79	Ever *vs.* never	1.33 (0.95–1.85)	Adjusted for period of interview, body mass index, age at menarche, age at menopause, parity, and oral contraceptive use, when appropriate.

Abbreviations: Na: not available; BMI: Body Mass Index.

### 3.2. Breastfeeding and Endometrial Cancer

**Figure 2:**
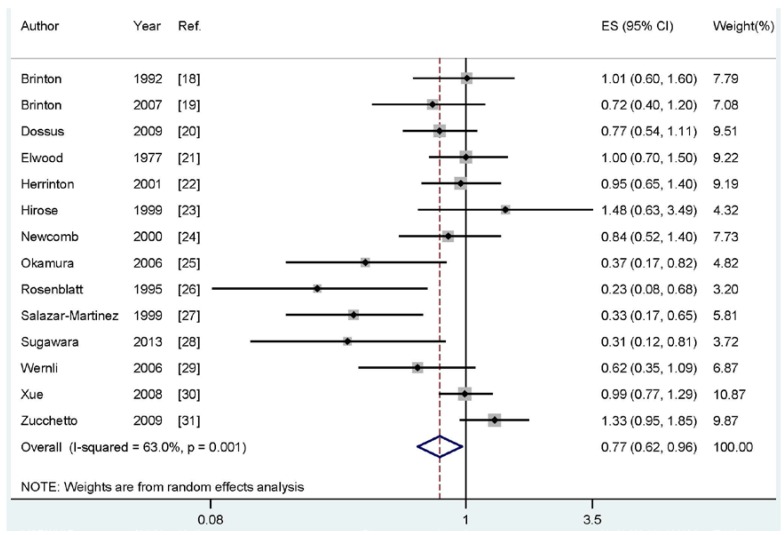
The forest plot of the association between breastfeeding and endometrial cancer risk.

Four of the included studies reported an inverse association between breastfeeding and endometrial cancer risk, while no significant association was reported in 10 studies. Our pooled results suggested that breastfeeding is inversely associated with the risk of endometrial cancer (summary RR: 0.77, 95% CI: 0.62–0.96, I^2^: 63.0%) (Figure 2).

### 3.3. Ever vs. Never Breastfeeding and Risk of Endometrial Cancer

Six studies [20,23,24,27,30,33] were included to investigated the association between ever breastfeeding *vs.* never breastfeeding and endometrial cancer risk. The summary RR of endometrial cancer for the ever breastfeeding compared with never breastfeeding was 0.85 (95% CI: 0.61–1.20; I^2^: 66.1%).

### 3.4. Longest Compared with Shortest Total Durations of Breastfeeding

Eight studies [21,22,25,26,28,29,31,32] involving 3422 endometrial cancer cases assessed the association between duration of breastfeeding and the risk of endometrial cancer. The summary RR of endometrial cancer risk for the longest category compared with shortest category of breastfeeding was 0.71 (95% CI: 0.53–0.95, I^2^: 60.3%).

### 3.5. Meta-Regression and Subgroups Analysis

As shown in Figure 2, evidence of heterogeneity (I^2^: 63.0%, *P*_heterogeneity_ = 0.001) was found in our pooled analysis. In order to explore the moderate between-study heterogeneity, univariate meta-regression with the covariates of publication year, geographic locations, study type (ever *vs.* never or longest *vs.* shortest), study design (retrospective or prospective), number of cases and source of controls were performed. However, no significant findings were found in the above-mentioned analyses.

When we conducted the subgroup analysis by study design, the association was significant in the retrospective studies (summary RR: 0.78, 95% CI: 0.58–0.98), but not in the prospective studies. In subgroup analysis for geographic locations, breastfeeding was significantly associated with reduced the risk of endometrial cancer in North America (summary RR: 0.87, 95% CI: 0.79–0.95), but not in Europe and Asia. Subgroup analysis by sources of control suggested that there is no significant association in the population-based retrospective studies or hospital-based retrospective studies. Furthermore, there is only one study involving seven cases that reported a breastfeeding duration of more than six years. Considering most studies reported the longest breastfeeding duration is about two years, we conducted a subgroup for those studies duration at two years. The pooled RR was 0.79 (95% CI: 0.59–0.98). The details results are summarized in Table 2.

**Table 2:** Summary risk estimates of the association between breastfeeding and endometrial cancer risk.

Sub-Groups	Cases	Studies	RR (95%CI)	I^2^ (%)	*P*_heterogeneity_
All studies	5158	14	0.77 (0.62–0.96)	63.0	0.001
**Study Design**					
Retrospective	3195	10	0.78 (0.58–0.98)	67.6	0.001
Prospective	1963	4	0.74 (0.52–1.04)	57.5	0.070
**Study Type**					
Ever *vs.* never	1736	6	0.85 (0.61–1.20)	66.1	0.011
Longest *vs.* shortest	3422	8	0.71 (0.53–0.95)	60.3	0.014
**Geographic Locations**					
North America	2474	6	0.87 (0.79–0.95)	48.8	0.082
Europe	2022	3	0.93 (0.62–1.40)	67.5	0.046
Asia	526	4	0.58 (0.31–1.07)	60.3	0.056
**Sources of Control**					
Population-based	1731	5	0.80 (0.57–1.11)	57.9	0.050
Hospital-based	1464	5	0.74 (0.41–1.34)	77.3	0.001
**Adjusted or Unadjusted Analyses**					
Adjusted results	4748	13	0.75 (0.59–0.95)	65.3	0.001
Unadjusted results	2966	5	0.83 (0.70–0.99)	15.2	0.318

### 3.6. Dose-Response Analysis

**Figure 3:**
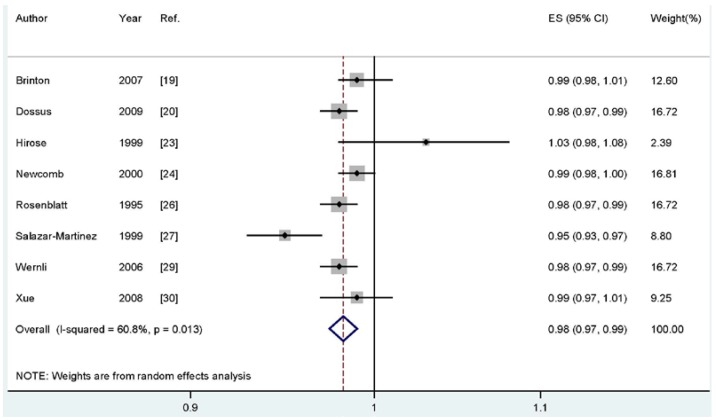
Dose-response meta-analyses of every one-month increase of breastfeeding and the risk of endometrial cancer. Squares represent study-specific RR, horizontal lines represent 95% CI and diamonds represent summary relative risk.

For dose-response analysis, eight studies [21,22,25,26,28,29,31,32] including 3422 cases were used for duration of breastfeeding and endometrial cancer risk. We found no evidence of statistically significant departure from linearity (*P*_for nonlinearity_ = 0.14). Our dose-response results indicated that increasing breastfeeding duration by one month was significantly associated with a 2% decrease in developing endometrial cancer risk (summary RR; 0.98, 95% CI: 0.97–0.99) (Figure 3).

### 3.7. Sensitivity Analysis and Publication Bias

**Figure 4:**
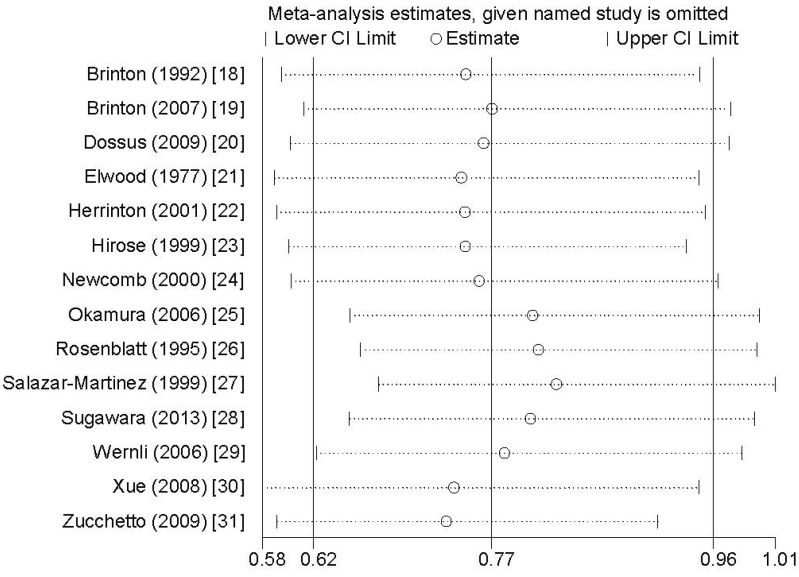
Analysis of influence of individual study on the association between breastfeeding and endometrial cancer risk. Open circles represent the pooled RR and horizontal lines represent the 95% CIs.

Sensitivity analysis showed that no individual study exerted excessive influence on the association between breastfeeding and endometrial cancer risk (Figure 4). Egger’s test (*p* = 0.168) showed no evidence of significant publication bias on the association between breastfeeding and endometrial cancer risk.

## 4. Discussion

The findings from this meta-analysis of epidemiologic studies indicated that breastfeeding could reduce the risk of endometrial cancer. The inverse association was also found for the longest compared with shortest categories of breastfeeding.

Breastfeeding has been suggested to reduce endometrial cancer risk, possibly because estrogen levels are low during lactation [27]. Most of this study finding can be interpreted on the basis of the “unopposed estrogen” hypothesis [3]. Furthermore, the mechanism by which breastfeeding could plausibly influence cancer risk is through the hormonal influence of the associated period of amenorrhea and infertility. In addition to the hormonal influence, the strong exfoliation of breast tissue during lactation and the massive epithelial apoptosis at the end of breastfeeding could contribute to decrease in the risk for cancer by eliminating cells with potentially unfavorable initial DNA damage [34]. These observations are generally consistent with our results.

Previous study had indicated that between-study heterogeneity is common in meta-analyses [35], and exploring the potential sources of between-study heterogeneity is an essential component of meta-analysis. For breastfeeding and the risk of endometrial cancer, moderate heterogeneity (I^2^: 63.0%, *P*_heterogeneity_ = 0.001) was found in the pooled results. The between-study heterogeneity might arise from publication year, geographic locations, study design (retrospective or prospective), study type (ever *vs.* never or longest *vs.* shortest duration), number of cases and source of controls. Thus, we used meta-regression to explore the causes of heterogeneity for covariates. However, no covariate had a significant impact on between-study heterogeneity for the above-mentioned covariates. Subgroup analyses by the study design (retrospective or prospective), study type (ever *vs.* never or longest *vs.* shortest duration), geographic locations and sources of controls were also conducted to explore the source of heterogeneity. However, the between-study heterogeneity persisted in some of the subgroups analyses. Endometrial cancer is a complex etiology and pathophysiology disease generated by the combined effects of genes and environment factors. Thus, other environment variables, as well as their possible interaction, may well be potential contributors to the heterogeneity observed.

As a meta-analysis of published studies, our findings showed some advantages. To our knowledge, this is the first comprehensive dose-response meta-analysis of breastfeeding and endometrial cancer risk. Second, we included large number of cases and participants, allowing a much greater possibility of reaching reasonable conclusions between breastfeeding and endometrial cancer risk. However, some limitations in this meta-analysis should be addressed. First, as a meta-analysis of observational studies, it was prone to recall or selection bias inherent in the original studies, especially in case-control studies. The information on exposures for prospective study is collected before the diagnosis of the disease, so that it is less susceptible to bias than retrospective studies. The results of the meta-regression showed no evidence of significant heterogeneity between subgroups, but the summary RR was different in subgroup analyses by study design. In our meta-analysis, the significant association was only found in retrospective studies, but not in the prospective studies, while only four studies included were prospective design. More original studies with prospective design would be beneficial in the future. Second, some individual studies did not adjust for potential confounders, which may have introduced bias in an unpredictable direction. Ever breastfeeding and the duration of breastfeeding are often associated with lower levels of BMI [36], a lower prevalence of OC use [37], and a lower prevalence of smoking [38]. Further studies should adjust for these factors. Third, there is only one study involving seven cases reporting breastfeeding duration of more than six years. Considering most studies reported the longest breastfeeding duration is about two years, we conducted a subgroup for those studies duration at two years. The pooled RR was 0.79 (95% CI: 0.59–0.98). The result of subgroup analysis is consistent with our overall results. Finally, no significant association was found for ever breastfeeding compared with never breastfeeding, and we could not extract the definitions of “ever breastfeeding” from each original study. The problem is that women who only ever make one attempt (and then fail) are classified in some databases as having breastfed. This happens in prospective data where midwives enter the data, and want to show that their units have high breastfeeding rates. This might be the reason why only the duration analysis was significant.

## 5. Conclusions

In summary, results from this meta-analysis suggested that breastfeeding, particularly a longer duration of breastfeeding, was inversely associated with risk of endometrial cancer. Dose-response analysis indicated that the risk reduced in endometrial cancer estimated is 2% for every one month increase in the duration of breastfeeding. In our study, we found a significant association between breastfeeding and endometrial cancer in North America, but not in Europe and Asia, probably due to the small number of cases included. Due to this limitation, further studies originating in other countries are required to assess the association between breastfeeding and endometrial cancer risk.

## Supplementary Files

Supplementary File 1
