# Supplementary material for: Association between Breastfeeding and Endometrial Cancer Risk: Evidence from a Systematic Review and Meta-Analysis
Source: Nutrients. 2015 Jul 14;7(7):5697–711. doi: 10.3390/nu7075248 (PMC4517025; doi:10.3390/nu7075248)
Supplement: Supplementary File 1 [file nutrients-07-05248-s001.docx]

Supplementary Materials

There are 29 studies [1–29] did not reported the RRs of the association between breastfeeding and endometrial cancer risk.

1. Hulka, B.S.; Brinton, L.A. Hormones and breast and endometrial cancers: Preventive strategies and future research. *Environ. Health Perspect.* **1995**, *103* (Suppl. 8), 185–189.
2. Kalandidi, A.; Tzonou, A.; Lipworth, L.; Gamatsi, I.; Filippa, D.; Trichopoulos, D. A case-control study of endometrial cancer in relation to reproductive, somatometric, and life-style variables. *Oncology* **1996**, *53*, 354–359.
3. McPherson, C.P.; Sellers, T.A.; Potter, J.D.; Bostick, R.M.; Folsom, A.R. Reproductive factors and risk of endometrial cancer: The Iowa women’s health study. *Am. J. Epidemiol.* **1996**, *143*, 1195–1202.
4. Fornasarig, M.; Campagnutta, E.; Talamini, R.; Franceschi, S.; Boz, G.; Scarabelli, C.; Andreaus, C.M.; Scozzari, G.; Valentini, M. Risk factors for endometrial cancer according to familial susceptibility. *Int. J. Cancer* **1998**, *77*, 29–32.
5. Parazzini, F.; Negri, E.; La Vecchia, C.; Benzi, G.; Chiaffarino, F.; Polatti, A.; Francheschi, S.
   Role of reproductive factors on the risk of endometrial cancer. *Int. J. Cancer* **1998**, *76*, 784–786.
6. Shields, T.S.; Weiss, N.S.; Voigt, L.F.; Beresford, S.A. The additional risk of endometrial cancer associated with unopposed estrogen use in women with other risk factors. *Epidemiology* **1999**, *10*, 733–738.
7. Terry, P.; Baron, J.A.; Weiderpass, E.; Yuen, J.; Lichtenstein, P.; Nyren, O. Lifestyle and endometrial cancer risk: A cohort study from the Swedish Twin Registry. *Int. J. Cancer* **1999**, *82*, 38–42.
8. Parslov, M.; Lidegaard, O.; Klintorp, S.; Pedersen, B.; Jonsson, L.; Eriksen, P.S.; Ottesen, B.
   Risk factors among young women with endometrial cancer: A Danish case-control study. *Am. J. Obstet. Gynecol.* **2000**, *182*, 23–29.
9. Mogren, I.; Stenlund, H.; Hogberg, U. Long-term impact of reproductive factors on the risk of cervical, endometrial, ovarian and breast cancer. *Acta Oncol.* **2001**, *40*, 849–854.
10. Hinkula, M.; Pukkala, E.; Kyyronen, P.; Kauppila, A. Grand multiparity and incidence of endometrial cancer: A population-based study in Finland. *Int. J. Cancer* **2002**, *98*, 912–915.
11. Horn-Ross, P.L.; John, E.M.; Canchola, A.J.; Stewart, S.L.; Lee, M.M. Phytoestrogen intake and endometrial cancer risk. *J. Natl. Cancer Inst.* **2003**, *95*, 1158–1164.
12. Xu, W.H.; Xiang, Y.B.; Ruan, Z.X.; Zheng, W.; Cheng, J.R.; Dai, Q.; Gao, Y.T.; Shu, X.O. Menstrual and reproductive factors and endometrial cancer risk: Results from a population-based case-control study in urban Shanghai. *Int. J. Cancer* **2004**, *108*, 613–619.
13. Hemminki, K.; Bermejo, J.L.; Granstrom, C. Endometrial cancer: Population attributable risks from reproductive, familial and socioeconomic factors. *Eur. J. Cancer* **2005**, *41*, 2155–2159.
14. Soliman, P.T.; Oh, J.C.; Schmeler, K.M.; Sun, C.C.; Slomovitz, B.M.; Gershenson, D.M.; Burke, T.W.; Lu, K.H. Risk factors for young premenopausal women with endometrial cancer. *Obstet. Gynecol.* **2005**, *105*, 575–580.
15. Setiawan, V.W.; Pike, M.C.; Kolonel, L.N.; Nomura, A.M.; Goodman, M.T.; Henderson, B.E. Racial/ethnic differences in endometrial cancer risk: The multiethnic cohort study. *Am. J. Epidemiol.* **2007**, *165*, 262–270.
16. Sherman, M.E.; Madigan, M.P.; Lacey, J.V., Jr.; Garcia-Closas, M.; Potischman, N.; Carreon, J.D.; Hartge, P.; Brinton, L.A. Ovarian volumes among women with endometrial carcinoma: Associations with risk factors and serum hormones. *Gynecol. Oncol.* **2007**, *107*, 431–435.
17. Vernaeve, V.; Bodri, D.; Colodron, M.; Vidal, R.; Durban, M.; Coll, O. Endometrial receptivity after oocyte donation in recipients with a history of chemotherapy and/or radiotherapy. *Hum. Reprod.* **2007**, *22*, 2863–2867.
18. Chavez-MacGregor, M.; van Gils, C.H.; van der Schouw, Y.T.; Monninkhof, E.; van Noord, P.A.; Peeters, P.H. Lifetime cumulative number of menstrual cycles and serum sex hormone levels in postmenopausal women. *Breast Cancer Res. Treat.* **2008**, *108*, 101–112.
19. Epplein, M.; Reed, S.D.; Voigt, L.F.; Newton, K.M.; Holt, V.L.; Weiss, N.S. Risk of complex and atypical endometrial hyperplasia in relation to anthropometric measures and reproductive history. *Am. J. Epidemiol.* **2008**, *168*, 563–570.
20. Linkov, F.; Taioli, E. Factors influencing endometrial cancer mortality: The Western Pennsylvania Registry. *Future Oncol.* **2008**, *4*, 857–865.
21. Reis, N.; Beji, N.K. Risk factors for endometrial cancer in Turkish women: Results from a
    hospital-based case-control study. *Eur. J. Oncol. Nurs.* **2009**, *13*, 122–127.
22. Schindler, A.E. Progestogen deficiency and endometrial cancer risk. *Maturitas* **2009**, *62*, 334–337.
23. Karageorgi, S.; Hankinson, S.E.; Kraft, P.; De Vivo, I. Reproductive factors and postmenopausal hormone use in relation to endometrial cancer risk in the Nurses’ Health Study cohort 1976–2004. *Int. J. Cancer* **2010**, *126*, 208–216.
24. Merritt, M.A.; Cramer, D.W. Molecular pathogenesis of endometrial and ovarian cancer.
    *Cancer Biomark.* **2010**, *9*, 287–305.
25. Milne, F.H.; Judge, D.S.; Preen, D.B.; Weinstein, P. Early life environment, life history and risk of endometrial cancer. *Med. Hypotheses* **2011**, *77*, 626–632.
26. Pocobelli, G.; Doherty, J.A.; Voigt, L.F.; Beresford, S.A.; Hill, D.A.; Chen, C.; Rossing, M.A.; Holmes, R.S.; Noor, Z.S.; Weiss, N.S. Pregnancy history and risk of endometrial cancer. *Epidemiology* **2011**, *22*, 638–645.
27. Amankwah, E.K.; Friedenreich, C.M.; Magliocco, A.M.; Brant, R.; Speidel, T.; Rahman, W.;
    Cook, L.S. Hormonal and reproductive risk factors for sporadic microsatellite stable and unstable endometrial tumors. *Cancer Epidemiol. Biomark. Prev.* **2013**, *22*, 1325–1331.
28. Dallal, C.M.; Brinton, L.A.; Bauer, D.C.; Buist, D.S.; Cauley, J.A.; Hue, T.F.; Lacroix, A.;
    Tice, J.A.; Chia, V.M.; Falk, R.; *et al*. Obesity-related hormones and endometrial cancer among postmenopausal women: A nested case-control study within the B~FIT cohort. *Endocr. Relat. Cancer* **2013**, *20*, 151–160.
29. Ali, A.T. Reproductive factors and the risk of endometrial cancer. *Int. J. Gynecol. Cancer* **2014**, *24*, 384–393.

© 2015 by the authors; licensee MDPI, Basel, Switzerland. This article is an open access article distributed under the terms and conditions of the Creative Commons Attribution license (http://creativecommons.org/licenses/by/4.0/).
